# Evolution of lineage-specific functions in ancient *cis*-regulatory modules

**DOI:** 10.1098/rsob.150079

**Published:** 2015-11-04

**Authors:** Stefan Pauls, Debbie K. Goode, Libero Petrone, Paola Oliveri, Greg Elgar

**Affiliations:** 1 Division of Systems Biology, Francis Crick Institute, Mill Hill laboratories, The Ridgeway, Mill Hill, London NW7 1AA, UK; 2 Cambridge Institute for Medical Research and the Wellcome Trust/MRC Cambridge Stem Cell Institute, University of Cambridge, Hills Road, Cambridge CB2 OXY, UK; 3 Department of Genetics, Evolution and Environment, University College London, Gower Street, London WC1 E6BT, UK

**Keywords:** *cis*-regulatory module, conserved non-coding element, zebrafish, enhancer evolution, *Sox21*

## Abstract

Morphological evolution is driven both by coding sequence variation and by changes in regulatory sequences. However, how *cis*-regulatory modules (CRMs) evolve to generate entirely novel expression domains is largely unknown. Here, we reconstruct the evolutionary history of a lens enhancer located within a CRM that not only predates the lens, a vertebrate innovation, but bilaterian animals in general. Alignments of orthologous sequences from different deuterostomes sub-divide the CRM into a deeply conserved core and a more divergent flanking region. We demonstrate that all deuterostome flanking regions, including invertebrate sequences, activate gene expression in the zebrafish lens through the same ancient cluster of activator sites. However, levels of gene expression vary between species due to the presence of repressor motifs in flanking region and core. These repressor motifs are responsible for the relatively weak enhancer activity of tetrapod flanking regions. Ray-finned fish, however, have gained two additional lineage-specific activator motifs which in combination with the ancient cluster of activators and the core constitute a potent lens enhancer. The exploitation and modification of existing regulatory potential in flanking regions but not in the highly conserved core might represent a more general model for the emergence of novel regulatory functions in complex CRMs.

## 1. Background

*Cis*-regulatory modules (CRMs) play a critical role in establishing complex and dynamic gene expression patterns in the embryo. In contrast to coding sequences, many CRM sequences are poorly conserved even between closely related species. The exception to this is a set of CRMs that we previously termed conserved non-coding elements (CNEs), which have been identified in all vertebrates from fish to mammals [1–4]. These sequences are clustered around developmental genes such as transcription factors and signalling molecules and, as shown in numerous reporter assays, are able to induce tissue-specific expression patterns during development [3,4].

CNEs provide a most valuable set of orthologous regulatory sequences for comparative studies that seek to understand the evolution of novel expression domains. However, in order to fully characterize regulatory changes that induce novel expression patterns in vertebrates it is vital to include ancestral sequences as well. Unfortunately, the vast majority of vertebrate CNEs are absent from invertebrates, which has led to the idea that they have evolved uniquely in vertebrates. However, recent results suggest that at least a handful of CNEs predate not only the emergence of vertebrates but possibly the emergence of bilaterian animals in general [5,6]. One vertebrate CNE in particular, linked to vertebrate *Sox21* genes, has also been identified in cephalochordates, echinoderms, hemichordates and cnidaria [5,7], indicating that, like coding sequences, some CRMs can have very long evolutionary histories.

The extraordinarily high sequence conservation across vertebrate CNEs suggests that these CRMs are largely inert to evolutionary change. Therefore, an important question is whether deeply conserved regulatory sequences still retain the plasticity to adopt new lineage-specific functions. Interestingly, it is known that the *Sox21* CNE referred to above has acquired at least one novel lineage-specific function in the vertebrate lens. Previous work has shown that, at least in fish, *Sox21* lens expression depends on this CNE (CNE17) and that in zebrafish *sox21b* is crucial for normal lens development [8]. Thus, the CRM defined by CNE17 predates the lens enhancer as well as the lens, which is generally considered a vertebrate innovation.

Little is known about how novel expression domains arise but currently there is evidence for at least four different modes of enhancer evolution. Some enhancers appear to evolve de novo from sequences showing no evidence of regulatory potential in the ancestral genome [9]. In other cases, insertions of mobile DNA sequences such as transposons have contributed to the emergence of novel expression domains at certain gene loci [10]. Chromosomal rearrangements can lead to displacement of boundary elements such as insulators after which a specific enhancer is allowed to interact with new target genes, a mechanism called promoter switching [11]. Finally, CRMs can be deployed into novel gene regulatory networks by co-option, as neatly demonstrated by recent findings in *Drosophila* [12]. The *Nep1* (*Neprilysin 1*) gene has a lineage-specific expression domain in the optic lobe restricted to *Drosophila santomea*. Despite the fact that the ancestral CRM only possesses a weak optic lobe enhancer it was found that optic lobe activity crucially depends on both ancient sequence motifs and lineage-specific changes. Cryptic enhancer activity has also been observed in evolutionary novelties over large evolutionary distances. For example, regulatory sequences from the *Ciona* homologue of vertebrate *beta-gamma-crystallins* can drive gene expression in the vertebrate lens despite the absence of this tissue in urochordates [13]. Therefore, two characteristic features seem to define enhancer co-option: the ancestral enhancer possesses cryptic enhancer activity in the new expression domain and lineage-specific changes in combination with ancestral motifs constitute the novel enhancer.

Here, we reconstruct the evolutionary history of the fish *Sox21* lens enhancer encoded in CNE17 using sequences from sea urchin, amphioxus, teleosts and mammals. We find that sequences from highly divergent species, including non-vertebrate deuterostomes, are all able to drive lens expression in developing zebrafish embryos. However, these sequences are not located in the deeply conserved core of the CNE but are situated immediately adjacent to it. In this flanking region, we identify an ancient cluster of sequence motifs functionally conserved from echinoderms to vertebrates that are crucial for lens activity. Within the deeply conserved core, we also identify an ancient repressor motif that modulates lens expression. Lastly, lineage-specific changes in fish have led to a de-repression of the lens enhancer. Therefore, the potential for evolutionary adaptability is retained in a deeply conserved CRM and novel enhancers can evolve in such a module by making use of pre-existing regulatory logic.

## 2. Results

**Figure 1.**
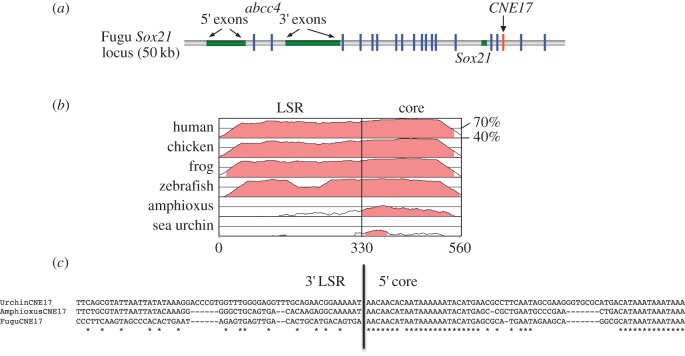
(*a*) Schematic of the *sox21* locus in Fugu showing the relative locations of the CNEs and coding sequences of the *abcc4* and *Sox21* genes (*Sox21* has just one coding exon). (*b*) VISTA plot of MLAGAN alignment between *Sox21* CNE17 from different species using Fugu as baseline. A vertical line indicates the division between LSR and core. (*c*) Multiple alignments across the boundary between the LSR and core regions using Fugu, amphioxus and sea urchin CNE17 sequences, highlighting the dramatic change in sequence identity.

An alignment of CNE17, which is located immediately downstream of the *Sox21*/*SoxB2* gene (figure 1*a*), across deuterostomes shows that the degree of sequence identity varies in different regions of the vertebrate CNE. Whereas at the 3′ end there is clearly identifiable sequence similarity between vertebrates and non-vertebrate deuterostomes, the 5′ end appears to be more lineage-specific, with only poor similarity between amphioxus and vertebrates and virtually none with sea urchin (figure 1*b*). We employed a series of extended pairwise and multiple alignments to accurately determine the boundary between the highly conserved ‘core’ region and the less conserved ‘lineage-specific region’ (LSR), and also examined the extent of sequence conservation 3′ of the core.

First, we generated a pairwise alignment between Fugu and amphioxus, the primary sequences used in this study. The LSR is defined by a rather weak alignment in the 5′ flank, with a sequence identity of 34% for 150 bp, rising to 45% from positions 151 to 330 (figure 1*b*). At this point, the sequence identity increases markedly to 74% for 80 bp followed by another 150 bp at 55% identity, after which the conservation drops to below 40% at around position 600. The two consecutive regions of high identity (74 and 55%) between amphioxus and Fugu define the core. Pairwise alignments between amphioxus and sea urchin, and Fugu and sea urchin present an almost identical de-lineation between 5′ LSR and highly conserved core. Multiple alignments between Fugu, amphioxus and sea urchin illustrate the clear change in sequence similarity across the boundaries of the LSR and core regions (figure 1*c*).

**Figure 2.**
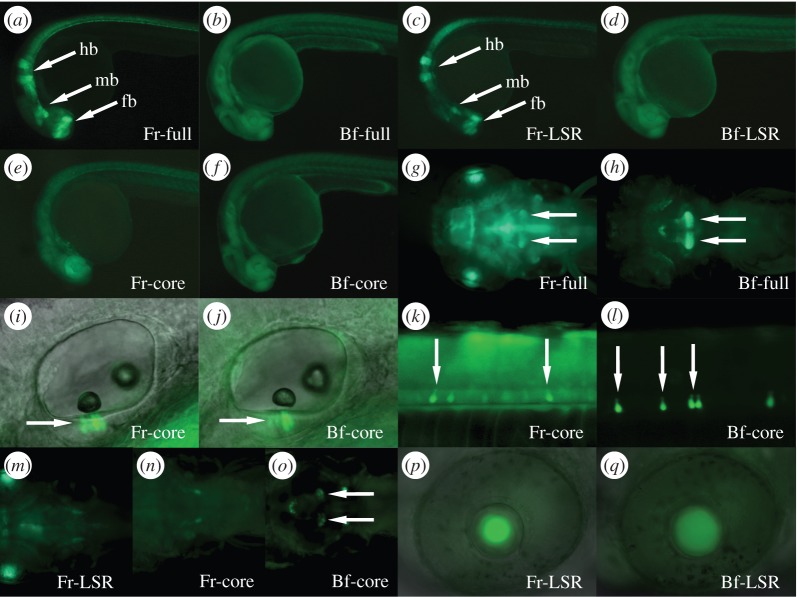
GFP expression in stable transgenic zebrafish lines induced by different parts of the Fugu (*a,c,e,g,i,k,m,n,p*) or amphioxus (*b,d,f,h,j,l,o,q*) CNE at 30 hpf (*a–f*), 50 hpf (*i–l,p,q*) and 5 dpf (*g,h,m,n,o*). Elevated levels of GFP in different brain regions (*a,c*) are indicated by arrows (fb, forebrain; mb, midbrain; hb, hindbrain). Arrows indicate expression in the hypothalamus (*g,h,o*), ear (*i,j*) and spinal cord KA-neurons (*k,l*).

We hypothesized that lineage-specific expression domains such as lens expression might be generated by the vertebrate LSR. To investigate this, we subdivided the Fugu and the amphioxus CNEs into LSR and core and established expression activation profiles for LSRs, cores and full-length CNEs from amphioxus and Fugu in stable transgenic zebrafish (figure 2).

The full-length Fugu element drives reporter expression in distinct regions of the brain (including the hypothalamus) as well as in the spinal cord and some sensory organs (figure 2*a,g*). While the Fugu LSR by itself defines some regional expression in the brain (figure 2*c*) and lens (figure 2*p*), the Fugu core region alone (figure 2*e*) can activate reporter expression in the sensory epithelial cells of the ear (figure 2*i*) and KA neurons in the spinal cord (figure 2*k*). By contrast, expression in the hypothalamus requires both LSR and core regions (figure 2*m,n*). The corresponding amphioxus full-length element drives more general and weaker expression in the brain and CNS (figure 2*b*) with the exception of the hypothalamus, where expression is clearly defined (figure 2*h*). Surprisingly, the amphioxus LSR is able to drive lens expression in zebrafish embryos but only when separated from the core (figure 2*q*). Apart from lens expression, the amphioxus LSR is unable to drive clear and specific expression in zebrafish embryos (figure 2*d*), while the core drives a similar pattern of reporter expression to the Fugu core region (figure 2*f,j,l*). Thus lens expression is driven by both vertebrate and amphioxus LSRs but in the amphioxus CNE, it is repressed by sequences in the core region (note the absence of lens expression from the full element in figure 2*h*).

In summary, and in agreement with previous studies [5,8], both the vertebrate and amphioxus CNEs activate widespread expression in the zebrafish CNS and sensory organs. These activation domains are also consistent with the endogenous expression of the vertebrate *Sox21* gene in the CNS and sensory organs [8,14–16] and more generally of the invertebrate *SoxB2* gene in embryonic neural tissue in sea urchin [5] and amphioxus [17]. Hence, these data strongly suggest that CNE17 is a multi-functional *SoxB2* CRM in a broad spectrum of organisms and that there appears to be some de-lineation of enhancer function between the core and LSR regions. In this study, we focus on lens expression, a lineage-specific function, which is driven by the vertebrate and amphioxus LSR (figure 2*p,q*).

**Figure 3.**
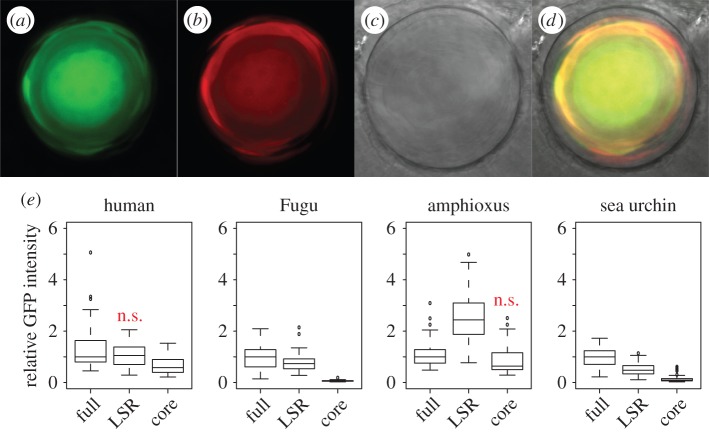
Quantification of GFP expression in the zebrafish lens. (*a–d*) Co-injection of CNE17::GFP reporter constructs (*a*) and an RFP lens standard (*b*) is used to measure relative GFP intensity in the lens at 52 hpf ((*c*, brightfield), (*d*, merged)). (*e*) Relative GFP intensity for full-length versions, LSR or core regions of CNE17 from different species. *p*-values Mann–Whitney test (electronic supplementary material, dataset S3), ‘n.s.’ = *p* > 0.05.

We used two different, semi-quantitative approaches to investigate conservation of lens enhancer activity in additional deuterostome orthologues of CNE17. In the first assay, we examined transient green fluorescent protein (GFP) expression in the zebrafish lens using a simple counting assay, after injecting single GFP constructs containing LSR and core regions from human, Fugu, amphioxus and sea urchin (electronic supplementary material, figure S1). We find that in all cases, including sea urchin, the LSR is sufficient for lens expression and also confirm that the complete amphioxus CNE17 is silent in the lens. In a second assay, also using transient *tol2* transgenesis in zebrafish, we co-injected a red fluorescent protein (RFP) standard together with the CNE17::GFP constructs in order to measure relative GFP fluorescence in the lens (figure 3*a–d*). Using this assay, we are able to show that the human and Fugu LSR contribute most of the lens activity to their respective full elements (figure 3*e*). For amphioxus, the LSR drives significantly more lens expression than the full element, and while the full sea urchin element drives strongest lens expression the LSR alone is still able to drive reporter expression whereas the core does not (figure 3*e*). These experiments confirm the relative importance of the LSR for lens expression compared with the core and also demonstrate that human and amphioxus sequences are only weakly active in the zebrafish lens compared to those of Fugu or sea urchin (electronic supplementary material, figure S2). However, we also note that both the Fugu and in particular the sea urchin core regions contribute to enhancer output in the lens (figure 3*e*).

**Figure 4.**
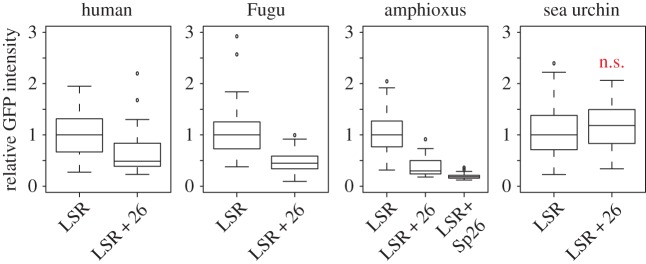
Conservation of 26 bp core repressor function in different sequences. GFP intensity relative to a co-injected RFP lens standard. The sea urchin 26 bp repressor does not inhibit the sea urchin LSR but represses the amphioxus LSR (LSR + Sp26). *p*-values Mann–Whitney test (electronic supplementary material, dataset S), ‘n.s.’ = *p* > 0.05.

The fact that lens activity driven by the amphioxus LSR is absent when the complete element is assayed suggests that the core region suppresses lens expression. We used a series of deletion and fusion constructs (electronic supplementary material, figure S3) to map a strong lens repressor to a 26 bp region immediately downstream of the LSR. Interestingly, this 26 bp motif is situated in the most highly conserved part of CNE17 and indeed we find that the equivalent 26 bp regions from both Fugu and sea urchin are also capable of repressing the amphioxus enhancer. Furthermore, this function appears to be conserved in the Fugu and human elements where the corresponding motifs also downregulate lens expression from the LSRs (figure 4). Interestingly, the sea urchin LSR seems to be inert to repression from this motif in terms of lens activity, yet when fused to the amphioxus LSR the sea urchin motif is an even more potent repressor than the amphioxus motif. This demonstrates that a region in the most highly conserved part of the CNE17 core sequence is negatively regulating lens expression driven by the LSR. It should be noted that removal of this motif alone does not de-repress the lens enhancer, suggesting that there are additional repressive sequences in the amphioxus core.

**Figure 5.**
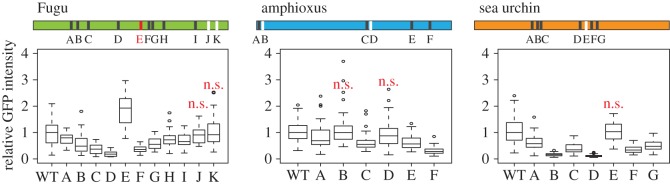
Requirement of Sox consensus motifs for lens expression. Sox consensus motifs in the Fugu, amphioxus and sea urchin LSR are labelled with capital letters. These motifs were individually mutated and, if required for lens expression, are shown in black. Motifs dispensable for lens expression are shown in white and the Fugu repressor motif in red. *p*-values Mann–Whitney test (electronic supplementary material, dataset S3), ‘n.s.’ = *p* > 0.05.

Previously, we found that lens activity regulated by another CNE at the *Sox21* locus crucially depends on a number of Sox consensus motifs [8]. Therefore, we considered whether the same class of sequence motifs might be involved in lens expression from CNE17. Indeed, we could identify a number of Sox consensus motifs in the LSRs from sea urchin, amphioxus and vertebrates (electronic supplementary material, figure S4). To determine their contribution to lens expression, we mutated each site independently and measured changes in relative GFP intensity compared with the WT sequence (figure 5; electronic supplementary material, figure S4). We find that most of the mutations have an impact on lens expression. In sea urchin, six out of seven consensus motifs appear to be crucial for lens expression and in amphioxus four out of six. In Fugu, there are 11 motifs that, with the exception of motif J, are also conserved in human. Mutations in eight of these motifs lead to a decrease in lens activity, two mutations do not seem to influence lens output and one mutation leads to an upregulation of the enhancer indicating a repressor motif. In summary, clusters of the same class of sequence motifs in the CNE17 LSR from sea urchin, amphioxus and vertebrates define enhancer activity in the vertebrate lens.

**Figure 6.**
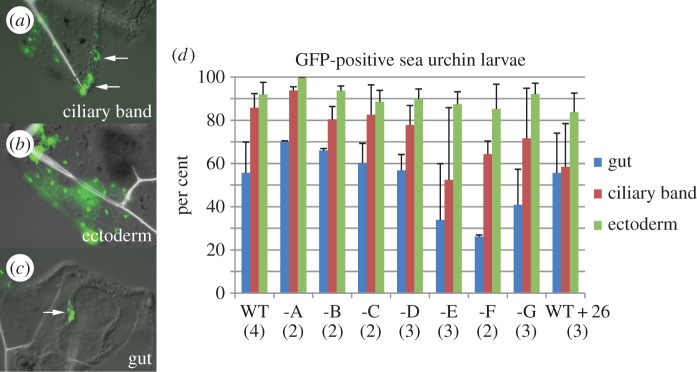
Requirement of Sox consensus motifs in sea urchin. (*a–c*) GFP expression in ciliary band cells (*a*), ectodermal cells (*b*) and gut cells (*c*) in 6-day-old sea urchin larvae. (*d*) Average percentage of larvae with GFP expression after the injection of the WT sea urchin LSR or constructs lacking the same Sox consensus motifs shown in figure 4. The last value (WT + 26) refers to the sea urchin LSR plus the 26 bp core repressor. Numbers in parentheses indicate independent injections. Error bars indicate standard deviation.

Conservation of a cluster of Sox motifs from sea urchin to vertebrates may suggest that it is also functional in sea urchin, albeit in a different context than the lens. Therefore, we tested the WT sea urchin sequence as well as constructs containing mutations in single Sox motifs in sea urchin. First, we wanted to identify tissues in which the sea urchin LSR would display strong enhancer activity. Using a vertebrate minimal *cfos* promoter together with the sea urchin LSR, we find GFP expression in cells of the ciliary band (a neurogenic structure in sea urchin) (figure 6*a*), the non-neurogenic (or aboral) ectoderm (figure 6*b*) and the gut (figure 6*c*). It has been shown previously that at this stage sea urchin *SoxB2* is expressed in ciliary band and gut [5] and that CNE17 in sea urchin can activate expression in the same two tissues [5]. However, the non-neurogenic ectoderm has not been reported to be a tissue expressing *SoxB2* at such a late stage or showing CNE17 enhancer activity. To rule out the possibility that ectoderm expression is induced by the vertebrate minimal promoter, we repeated the injections using the sea urchin minimal *endo16* promoter (electronic supplementary material, figure S5*a–d*) as well as the vertebrate *cfos* promoter on its own (electronic supplementary material, figure S5*e*). Despite being slightly less active than the *cfos* promoter the *endo16* construct was active in the same tissues including the ectoderm. Moreover, GFP expression induced by the *cfos* promoter on its own is negligible compared to when fused to the LSR. Therefore, we decided to use the stronger *cfos* promoter to investigate the impact of each Sox consensus motif on enhancer function. We assayed the WT LSR and each mutation independently by scoring larvae with GFP expression in gut, ciliary band and ectoderm (figure 6*d*). We find that three mutations induce a change in enhancer activity in ciliary band and gut albeit to a different extent. Mutation of motif F consistently leads to a reduction in the number of embryos with GFP expression in ciliary band cells or gut while changes in the two motifs to either side of F, that is E and G, increase variability of the enhancer output. This suggests that at least some of these motifs active in the vertebrate lens also participate in endogenous enhancer function in sea urchin. Moreover, although we established earlier that lens expression in vertebrates driven by the sea urchin LSR is not suppressed by the 26 bp core repressor region (figure 4), in sea urchin expression in ciliary band cells is sensitive to this short repressor motif (figure 6*d*). These findings indicate functional conservation of the 26 bp repressor across deuterostomes and suggest that the cluster of Sox consensus motifs in the LSR of CNE17 is functional in echinoderms and vertebrates.

The weaker activity of the human LSR compared with the orthologous Fugu region (electronic supplementary material, figure S2) suggests lineage-specific differences in lens activity between fish and tetrapods. Therefore, we measured enhancer activity of a variety of vertebrate CNE17 sequences including a cartilaginous fish, the elephant shark (electronic supplementary material, figure S6*a,b*) in zebrafish. We find that the elephant shark sequence contains by far the strongest lens enhancer and that teleost sequences consistently perform better than tetrapod sequences. As described above, the Fugu LSR contains not only activating motifs but also one Sox consensus motif (E) (figure 5) with repressor function in the lens. One possible explanation for the strong lens enhancer in the elephant shark sequence would be the absence of this repressor function as is the case for invertebrate LSRs*.* To confirm that the repressor is not just restricted to ray-finned fish, we first targeted this motif in the human sequence (electronic supplementary material, figure S6*c*). This leads to a marked increase in lens expression and suggests that the repressor function is conserved in all bony vertebrates. Contrary to this, when targeting the same motif in the elephant shark LSR we see a small but significant loss in lens activity. This suggests that it does not function as a repressor in the chondrichthyes sequence, at least when tested in zebrafish, which may be the reason for the strong lens expression in zebrafish from the elephant shark sequence.

**Figure 7.**
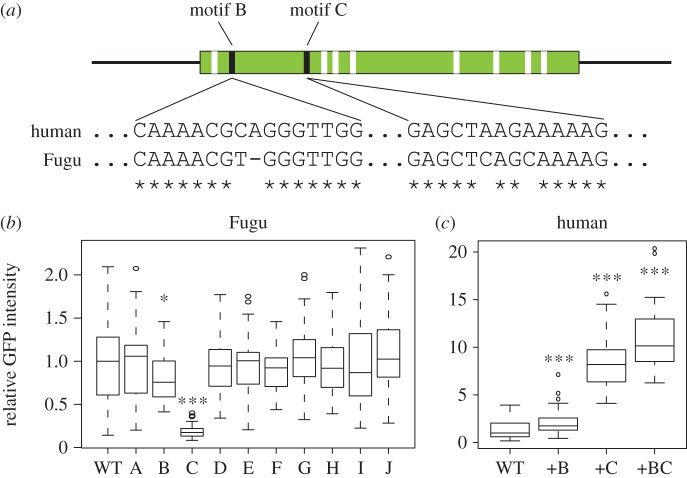
Fugu-specific motifs required for lens expression. (*a*) Minimal lens enhancer in the Fugu LSR. Motifs different from human are indicated in white if dispensable or black if crucial for lens expression. (*b,c*) Relative GFP intensity in the zebrafish lens after the injection of either Fugu CNE17 including human motifs A–J instead of the corresponding Fugu motif (*b*) or insertion of Fugu motifs B or C or both into the human LSR (*c*). *p*-values Mann–Whitney test (electronic supplementary material, dataset S3), *p < 0.05, ***p < 0.005.

The differential lens activity between teleost and tetrapod sequences suggests that additional lineage-specific changes have occurred in fish that are crucial to overcome repression. Therefore, we aligned the minimal lens enhancer region in the Fugu LSR (electronic supplementary material, figure S7) to the corresponding region in human and identified several differences which we clustered into 10 putative sequence motifs (electronic supplementary material, figure S8). Next, we targeted each of these motifs independently by introducing the corresponding human sequence into the Fugu LSR. Two of these changes lead to a decrease in enhancer activity (figure 7*a,b* and electronic supplementary material, figure S8). Loss of motif C in the Fugu sequence almost completely abolishes lens expression, whereas motif B has a weaker but nevertheless significant impact on enhancer function. To verify these findings, we performed the reciprocal experiment by introducing Fugu motifs B and C into the human sequence (figure 7*c*). As expected, we find a mild increase in lens activity on the introduction of Fugu motif B and a very strong increase on the introduction of motif C. The importance of motif B is further emphasized by the fact that a combination of Fugu B and C in the human sequence further increases enhancer output compared to motif C on its own. Alignments across all vertebrates show that both motifs are specific to ray-finned fish (electronic supplementary material, figure S9) and not just teleosts, as they are also present in spotted gar. Thus, the Fugu lens enhancer depends both on an ancient cluster of sequence motifs probably dating back to before the emergence of chordates and on lineage-specific changes in ray-finned fish.

## 3. Discussion

**Figure 8.**
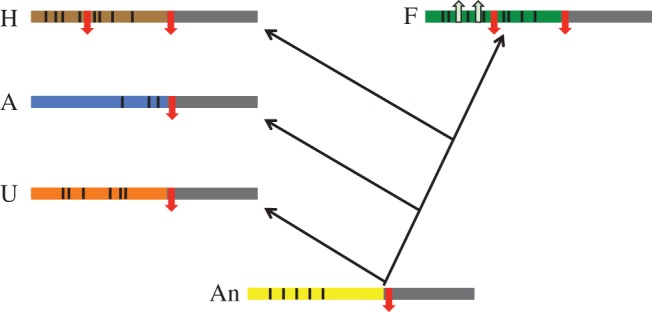
A model for lens enhancer evolution in CNE17. See main text for details. Core regions are shown in dark grey with the putative ancestral (An) LSR in yellow. Sox consensus motifs are shown as black lines in the LSRs (a cluster of these motifs is assumed to be already present in the ancestral LSR). Repressor motifs are shown as downward red arrows and lineage-specific activators as upward grey arrows. H, human; A, amphioxus; U, sea urchin; F, Fugu.

In this study, we have recapitulated the evolution of a vertebrate-specific lens enhancer embedded in a deeply conserved CRM. We note in the introduction that the criteria for the evolution of novel expression domains via co-option of enhancers are the presence of pre-existing cryptic enhancer activity in the novel domain encoded within the ancestral sequence, while robust expression is dependent on both ancestral and novel sequence motifs [12]. These criteria are met for the teleost lens enhancer. Our findings suggest a model in which the ancestral deuterostome CNE17 already possessed a cluster of Sox consensus motifs in its LSR as well as a repressor sequence in its core (figure 8). These Sox consensus motifs already confer at least basal levels of lens activity on LSRs from both invertebrate deuterostomes and vertebrates when tested in zebrafish. Moreover, this cluster, as well as the core repressor, is an ancient functional subunit of CNE17 as it influences regulatory output not only in zebrafish but also in sea urchin. However, the evolution of vertebrates is paralleled by the emergence of a potent lens repressor in the LSR, also resembling a Sox consensus motif, resulting in only low levels of lens activity despite the presence of the ancestral cluster of activator motifs. Fish, however, may have exploited putative cryptic lens activity in the ancestral sequence and acquired a strong lens enhancer in the LSR through the acquisition of two additional lineage-specific activator motifs. Therefore, robust output from the fish lens enhancer depends on both ancestral and lineage-specific regulatory features. A question arising from these findings is how widespread an evolutionary scenario this might be in terms of both a subdivision in LSR and core and the emergence of novel enhancers by deploying ancestral regulatory logic.

This pattern of enhancer evolution is reminiscent of another set of highly conserved CRMs. A survey of the lamprey genome identified at least a subset of vertebrate CNEs in this jawless vertebrate [18]. Interestingly, in this set high sequence identity is also restricted to only a sub-region of the CNE. This suggests a subdivision into ancestral cores and LSRs for either jawed or jawless vertebrates similar to CNE17. Despite the fact that nothing is known concerning the tissue-specificities of these flanks and cores, this pattern of conservation seems to be more widespread and presents an attractive model for the evolution of lineage-specific enhancers. After a CRM has reached a certain level of complexity, any mutation that might optimize one enhancer function in most cases would impede others. This may lead to the establishment of core regions that are relatively inert to evolutionary change. In fact, as we show, the functional output of a core can remain constant over huge evolutionary distances. However, new regulatory features can evolve in close proximity to a core and this perhaps may prove to be more common than evolution of novel isolated enhancers. A novel enhancer may benefit from an existing core by the fact that some general pioneer transcription factors important to open up the chromatin already bind to this area in the DNA. If this was the case initially flanking regions would depend on the core for proper gene activation. This dependency on the core would be likely to diminish over time as more and more regulatory factors bind to the flanking region, some of which may have pioneering functions on their own. However, we observe cross-talk between LSR and core for gene expression in the hypothalamus and in the lens. Hypothalamic expression from the Fugu CNE crucially depends on the presence of both LSR and core, and lens activity in the LSR is significantly amplified by the presence of a core. It is an attractive model to consider the core as a seed around which new regulatory features can emerge more easily than de novo.

Our detailed reconstruction of the evolution of the lens enhancer demonstrates that when deploying ancestral regulatory logic, very few lineage-specific changes are needed to generate novel expression domains. It is very likely that the strong lens enhancer in CNE17 has evolved only in ray-finned fish. In general, *Sox21* lens expression seems to be absent from vertebrates except for teleosts and chicken [8,14]. Moreover, unlike zebrafish [8], there is no evidence of a crucial role for *Sox21* during lens development in mouse [19]. This may explain why tetrapod sequences tested in zebrafish perform relatively poorly in the lens. A lens enhancer in CNE17 may have evolved independently in birds but, as we show, would rely on different lineage-specific activator motifs than in fish. Alternatively, a lens enhancer in birds could have evolved in a different CNE at the *Sox21* locus. By identifying the lineage-specific changes in the fish lens enhancer, we provide a starting point for the examination of how *Sox21* lens expression has evolved in different vertebrate lineages.

However, this enhancer does not only depend on lineage-specific motifs but also on ancestral motifs probably present in all deuterostomes. Developmental enhancers typically consist of a number of activating and repressing motifs to account for their complex and dynamic regulatory output. It is this number of binding sites that poses a particular problem to the evolution of these enhancers within existing CRMs. Insertion of a battery of novel sites via a transposable element into a CRM would disrupt the whole sequence and generation of multiple new sites through a number of mutations might have too huge an impact on other regulatory functions. The only way to generate a novel enhancer it seems is to exploit regulatory logic already embedded in the sequence. Such cryptic enhancer potential in a CRM might already induce low and unstable levels of gene expression before the emergence of the novel enhancer similar to the amphioxus or human LSRs in the zebrafish lens. Subsequently, this potential, in our case encoded by an ancestral cluster of Sox consensus motifs, can be exploited to form a novel enhancer. The fact that the Sox cluster is also functional in sea urchin raises the question of its ancestral function that has caused its conservation in vertebrates before its deployment in the fish lens enhancer. One possibility is a role during neurogenesis because *SoxB2* genes are expressed in embryonic neural tissue in sea urchin [5], amphioxus [17] and vertebrates [14–16] and in chicken a carefully regulated balance between *SoxB1* and *SoxB2* genes guides the production of neuronal cells from an undifferentiated precursor pool [20]. This may also hint at a role for the core repressor motif because establishing this balance would require activation as much as repression.

Our findings uncover several important aspects of the evolution of the CNE17 lens enhancer. Furthermore, we have discussed the possibility of these being more general principles underlying the evolution of complex developmental CRMs, if not regulatory sequence evolution in general. Another clear outcome of this study is that deeply conserved CRMs, even if their degree of sequence conservation may suggest differently, are by no means recalcitrant to functional change. Instead they can readily participate in the evolution of lineage-specific gene regulatory networks and provide a source of regulatory potential that can be exploited to evolve novel expression domains.

## 4. Material and methods

### 4.1. Genomic sequences and alignments

Fugu, mouse, human and orthologous non-vertebrate sequences for CNE17 correspond to previously identified genomic regions [3,5,7]. For other vertebrates, we used BLAT to detect CNE17 in elephant shark, zebrafish, *Xenopus tropicalis* and chicken. Coordinates of CNE regions, LSRs and cores used in the reporter assay are given in the electronic supplementary material, dataset S1. Multiple alignments, conservation profiles and VISTA plots were obtained by using MLAGAN (lagan.stanford.edu) with 60% identity over 50 bp as cut-off.

We used Clustal [21] alignments to accurately define the core and LSRs of CNE17.

### 4.2. Generation of reporter constructs and transgenic lines

Genomic coordinates for deletion or fusion constructs of CNE17 are listed in the electronic supplementary material, table S2. GFP reporter constructs were generated as described in Pauls *et al.* [8]. Briefly, sequences were PCR-amplified from genomic DNA and cloned into the pCR8/GW/TOPO TA cloning vector (Invitrogen). This served as an entry clone for inserting the PCR products into a GFP expression vector described in Fisher *et al.* [22] allowing for *tol2*-mediated transgenesis in zebrafish [23]. In cases where stable transgenic zebrafish lines were analysed, each expression domain was confirmed in at least three independent lines. The RFP standard used for quantification of GFP lens expression was generated by cloning a lens enhancer linked to zebrafish *sox2* (chromosome 22 : 40307301–40307520) and first identified in chicken [24]. The expression vector for the standard was generated by substituting the *GFP* sequence in the *tol2* expression vector from [22] with an *RFP* sequence (gift from Javier Terriente). Mutagenesis was conducted following the QuickChange protocol using PfuUltra (Agilent Technologies) and the *tol2* WT CNE clones as templates.

### 4.3. Detection of Sox consensus motifs

Sox consensus motifs assayed in this study were identified as described in Pauls *et al.* [8]. We used the JASPAR database and the implemented algorithm [25] to scan CNE17 regions from different species. More precisely we used the 'SOX10′ motif included in JASPAR as a generic Sox motif and a threshold of 0.89 for detection.

### 4.4. Sea urchin injections

Linearized DNA constructs of equal molar concentration were injected into sea urchin *Strongylocentrotus purpuratus* according to Cheers & Ettensohn [26]. WT or mutated versions of the sea urchin CNE were either fused to a *cfos* minimal promoter [22] or to a sea urchin *endo16* minimal promoter known to have negligible background activity without an enhancer sequence [27]. Every construct was injected at least twice into eggs obtained from different females. We screened an average of about 76 larvae for each construct (lowest number = 47, highest number 128).

### 4.5. Lens assays

Two transient lens assays were conducted during this study. In the first one, we injected GFP reporter constructs into zebrafish and counted the number of GFP-positive lenses in 52 hpf old embryos under a fluorescence stereomicroscope. In the second assay, an RFP lens standard was co-injected together with the GFP constructs and relative GFP intensities were determined in 52 hpf old zebrafish using a confocal microscope.

#### 4.5.1. Counting assay

Two transient lens assays were conducted during this study. In the first assay, we injected GFP reporter constructs into zebrafish eggs at the one-cell stage and counted the number of positive lenses in live embryos at around 52 hpf or more than 20 h after the onset of enhancer activity in the lens from the Fugu element [8]. This leaves enough time for the detection of a robust fluorescent signal in the lens. Prior to counting GFP-positive lenses, we discarded those embryos with the weakest GFP signal, but never more than 10% of the entire batch. We then randomly picked about 30–50 embryos (or 60–100 lenses) and determined GFP expression under a Leica MZ 16F dissecting microscope. The injections were repeated in total three times for each construct.

#### 4.5.2. Relative intensity assay

For the second assay measuring relative GFP intensity, we co-injected an RFP lens reporter alongside the GFP constructs. At around 52 hpf, we picked approximately 25 live embryos showing robust RFP expression, not considering GFP expression when selecting embryos, in at least one lens and mounted them for examination under a confocal microscope. This was repeated at least twice for each construct. We performed three scans sampling a 20 µm thick region corresponding to the central region of each lens. Owing to differences in lens activity, we used two different confocal settings when performing the scans. All scans for the amphioxus or human constructs (except for ‘Hs’ in the electronic supplementary material, figure S6*b*) were conducted reducing the laser output for scanning the RFP four times compared to all other scans. This was necessary to detect robust GFP signals when using identical gain settings. Mean fluorescent intensities in the lens were determined using ImageJ (http://imagej.nih.gov/ij/index.html). Next, we determined the mean intensity for a single lens by calculating the average for each trio of GFP and RFP scans of the same lens. In order to correct for unwanted position effects affecting any of two co-injected transgenes, we discarded the most extreme outliers in all lens scans. This was done first by calculating how much the GFP or RFP values for a single lens would diverge from the average GFP or RFP value of that particular set of scans. More precisely we calculated the absolute difference between a single measurement (sm) and the mean (m) divided by the mean [divergence = (|sm – m|)/m)]. Next we discarded the 7.5% most divergent GFP or RFP scans among all constructs tested assuming that those were the ones most likely to be affected by the site of integration into the genome. This reduced the variance-to-mean ratio for the relative intensity for all scans by a factor of 0.86 and left us with an average of 48.8 lenses scanned for each construct (minimum number = 30, maximum number = 139). For the boxplots showing the dispersion of the single measurements for one construct, we normalized all values by setting the median of the reference construct (always on the left in each plot) to one. This means the *Y*-axis is showing the ‘fold-change’ with respect to the median of the reference construct. Significant differences between the reference construct and other constructs in the same plot were detected using the Mann–Whitney test implemented in the R package (http://www.r-project.org/).

## Supplementary Material

Dataset1

## Supplementary Material

Dataset2

## Supplementary Material

Dataset3

## Supplementary Material

Supplementary_figures_1_to_9
