# Supplementary material for: Evolution of lineage-specific functions in ancient cis-regulatory modules
Source: Open Biol. 2015 Nov 4;5(11):150079. doi: 10.1098/rsob.150079 (PMC4680567; doi:10.1098/rsob.150079)
Supplement: Supplementary_figures_1_to_9 [file rsob150079supp4.pdf]

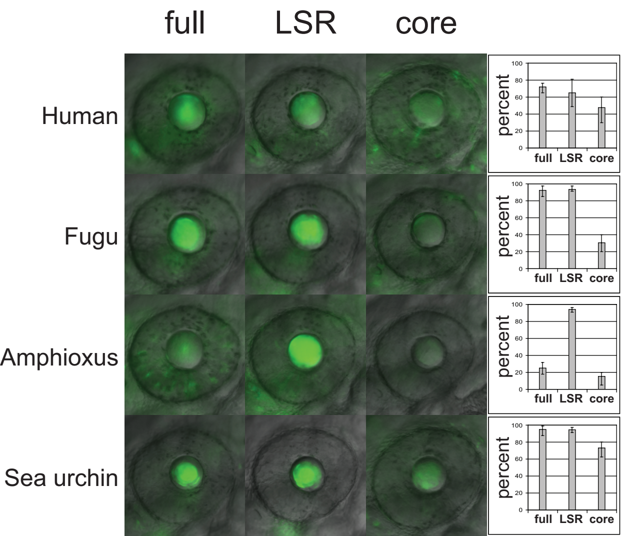

full
LSR
core
Human
Fugu
Amphioxus
Sea urchin
percent
0
20
40
60
80
100
full LSR core

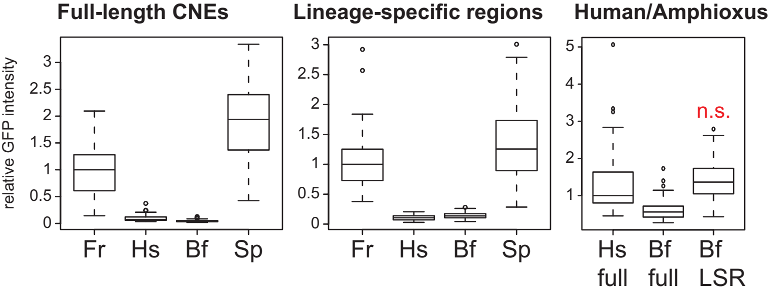

Full-length CNEs
Lineage-specific regions
Human/Amphioxus
relative GFP intensity
0
0.5
1
1.5
2
2.5
3
4
5
Fr
Hs
Bf
Sp
Hs full
Bf full
Bf LSR
n.s.

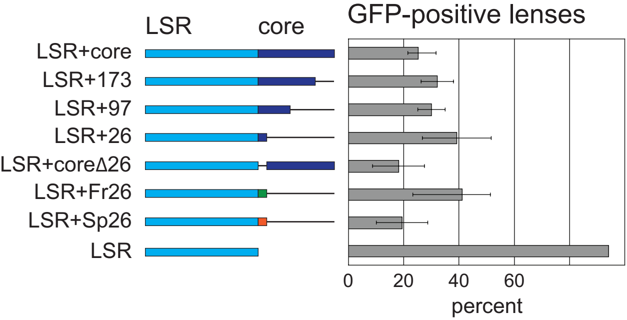
LSR
core
GFP-positive lenses
LSR+core
LSR+173
LSR+97
LSR+26
LSR+coreΔ26
LSR+Fr26
LSR+Sp26
LSR
0
20
40
60
percent

Fugu

TGGGAGCGTTTAGGCGGTCGAGGGGGTTTT
GTCTGGGAACAAAACGTGGGTTGGGAGGTT
TTGTGAGAGTGTTGTTTGTTGAAGTGGAGC
TCAGCAAAAGCGGCTGCTTTCCTTCATTGT
GATGAAAGCAATCAGTGGTATTTGGAAAAC
TGTTAGCATTGTGCACTTCTTCTGTGTCCG
TTGTGGAGGATTTCTTTTCACAAGGTTTTT
TCAGGCGATCCAGCTGGCCGGAGTGAATAG
CACTGCAATGTGTACACGCTTTGTCCCTCC
AAGCCCTTCAAGTAGCCCACACTGAATAGA
GTGAGTTGACACTGCATGACAG

A TTTTGT -> TTTCAT
B (TTTTGT) -> TTTCAT
C TTTTGT -> TTTCAT
D CATTGT -> CATGTT
E CATTGT -> CATCAT
F CTGTGT -> CTGCAT
G CGTTGT -> CGTCAT
H (CCTTGT) -> CCTCAT
I CTTTGT -> CTTCAT
J (CAGTGT) -> CAGCAT
K (CAGTGT) -> CAGCAT

Amphioxus

GTTGTTTGGACACTGTCTGCATTGTTATCC
GCCCCTAAATTGCTCTTAAGGCCGCCAGAT
GGATTCTAAAGTTTGTGTACAAATTACGAT
CGGGGTGCGAGTCTCCGAGCACCACACCAG
GTTTTACCTCTTTGCCTACAAGACCGTAGG
GTAGTGAGAGGGCTTAGGTACAATTGTGTC
GGGAGGGGGCTATTTTGTCCGTTGTGTGTA
GTGTTCCCTTTCAGCAGCCATACAGCTGGG
GGAGTTGAATGGGTTCGCCGCTGTAGGCCT
CCATTGTTCCCGCTTGTTCTGCGTATTAAT
TATACAAAGGGGGCTGCAGTGACACAAGAG
GCAAAA

A (CAGTGT) -> CAGCAT
B CATTGT -> CATCAT
C TTTTGT -> TTTCAT
D CGTTGT -> CGTCAT
E CATTGT -> CATCAT
F (CTTTGT) -> CTTCAT

Sea urchin

GGGCACTTCTCCTTGATACGTTGGTTGGTG
TTCATGACCACCCCCCCCCCCCCTCTCCTT
TCGGCTTTCCTCTTTCCCTCTCCATTTCGC
TATTGAAATAGCCACGCATTGAGCTTTGTC
ATTGCTTTCTATTTTGTGTTGCCTGAATAG
CCGACTATGATAGTCTTTGCATTCTTCGAG
AATTTACATTCTATTCATGGCGAGGTGGCC
ATTGTACCTGGCTGTGTGTTGCCATTGTGA
AGGACAAAAGGCACTGCAAACCACTTTTGA
ATAGCCCCAACTTCGCAAGCACTAAGTCCC
TTTCAATGCTATTGTCCAAGCTTGTTCAGC
GTATTAATTATATAAAGGACCCGTGGTTTG
GGGAGGTTTGCAAAACGGAAAAATAACAAC

A CTTTGT -> CTTCAT
B CTTTCT -> CTTCAT
C TTTTGT -> TTTCAT
D CATTGT -> CATCAT
E CTGTGT -> CTGCAT
F CATTGT -> CATCAT
G (TTTTGT) -> TTTCAT

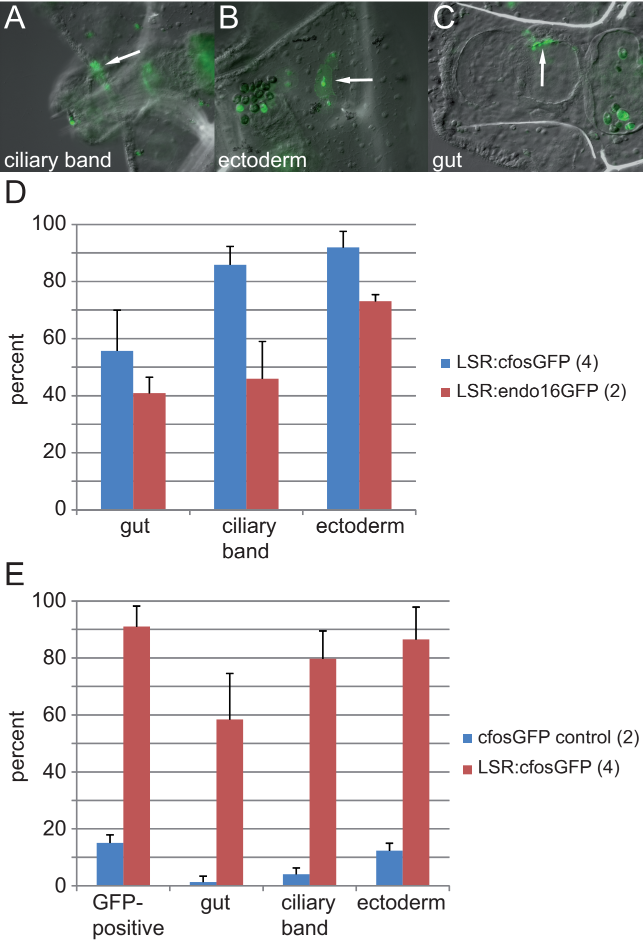
A
ciliary band
B
ectoderm
C
gut
D
percent
100
80
60
40
20
0
gut
ciliary band
ectoderm
LSR:cfosGFP (4)
LSR:endo16GFP (2)
E
percent
100
80
60
40
20
0
GFP-positive
gut
ciliary band
ectoderm
cfosGFP control (2)
LSR:cfosGFP (4)

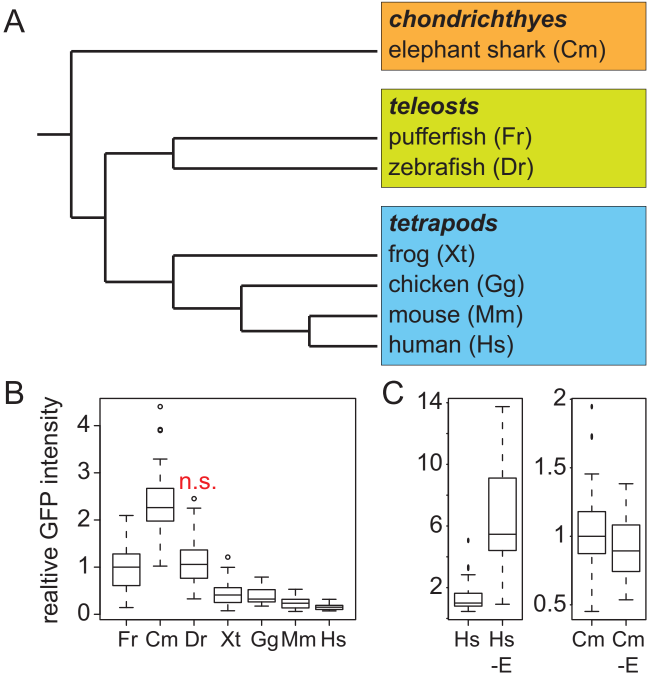
A
chondrichthyes
elephant shark (Cm)
teleosts
pufferfish (Fr)
zebrafish (Dr)
tetrapods
frog (Xt)
chicken (Gg)
mouse (Mm)
human (Hs)
B
realtive GFP intensity
0
1
2
3
4
n.s.
Fr Cm Dr Xt Gg Mm Hs
C
14
10
6
2
Hs Hs -E
2
1.5
1
0.5
Cm Cm -E

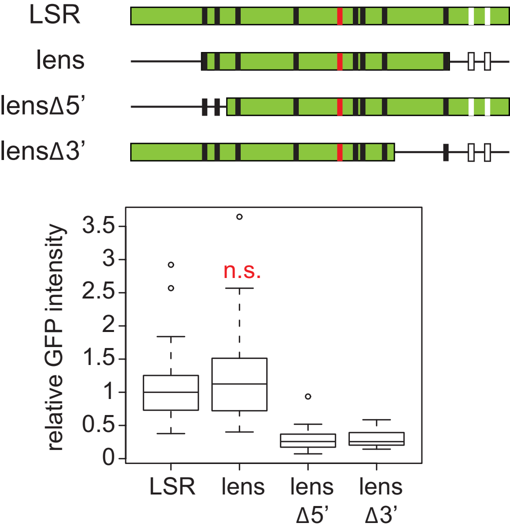
LSR
lens
lensΔ5’
lensΔ3’
relative GFP intensity
3.5
3
2.5
2
1.5
1
0.5
0
n.s.
LSR
lens
lens Δ5’
lens Δ3’

Fugu

TTTTGTC**T**G**G**GAACAAAACG**T-**GGGTTGGGAGGTTTTGTGAGAGTG
TTGTTTGTTGAAGTGGAGCT**C**AG**C**AAAAGCGGC**T**GCTTTC**-**C**T**TCA
TTGTGA**T**GAAA**G**CAATCAGTGGTATTTGGAAAACTGTTAGCATTGT
GCACTTCTTCTGTGTCCGTTGTG**GA**G**GA**TTTCTTTTCACAAGGTTT
TTT**---**CAGGCGATCCAGCTGGCCGGA**G**TGAATAGC**AC**TGCAATGT
GTACACGCTTTGT

A:	GTC**T**G**G**GAA	->	GTC**C**G**A**GAA
B:	ACG**T-**GGG	->	ACG**CA**GGG
C:	GCT**C**AG**C**AAA	->	GCT**A**AG**A**AAA
D:	GGC**T**GCT	->	GGC**G**GCT
E:	TTC**-**C**T**TCA	->	TTC**T**C**C**TCA
F:	TGA**T**GAAA**G**CAA	->	TGA**A**GAAA**C**CAA
G:	GTG**GA**G**GA**TTT	->	GTG**AG**G**CG**TTT
H:	TTT**---**CAG	->	TTT**TTT**CAG
I:	GGA**G**TGA	->	GGA**A**TGA
J:	AGC**AC**TGC	->	AGC**GG**TGC

	MOTIF B	MOTIF C
**Human**	AAAACG**CA**GGGTTG	GGAGCT**A**AG**A**AAAA
**Macaque**	AAAACG**CA**GGGTTG	GGAGCT**A**AG**A**AAAA
**Mouse**	AAAACG**CG**GGGTTG	GGAGCT**A**AG**A**AAAA
**Rabbit**	AAAACG**CA**GGGTTG	GCAGCT**A**AG**A**AAAA
**Dog**	AAAACG**CA**GGGTTG	GGAGCT**A**AG**A**AAAA
**Horse**	AAAACG**CA**GGGTTG	GGAGCT**A**AG**A**AAAA
**Dolphin**	AAAACG**CA**GGGTTG	GGAGCT**A**AG**A**AAAA
**Pig**	AAAACG**CA**GGGTTG	GGAGCT**A**AG**A**AAAA
**Megabat**	AAAACG**CA**GGGTTG	GGAGCT**A**AG**A**AAAA
**Elephant**	AAAACG**CA**GGGTTG	GGAGCT**A**AG**A**AAAA
**Hyrax**	AAAACG**CA**GGGTTG	GCAGCT**A**AG**A**AAAA
**Lesser Hedgehog**	AAAACG**TA**GGGTTG	GGAGTT**A**AG**A**AAAA
**Armadillo**	AAAACG**CA**GGGTTG	GGAGCT**A**AG**A**AAAA
**Opossum**	AAAACG**CA**GGGTTG	GGAGGT**A**AG**A**AAAA
**Platypus**	AAAACG**CA**GGGTTG	GGAGCT**A**AG**A**AAAA
**Tasmanian devil**	AAAACG**CA**GGGTTG	GGAGCT**A**AG**A**AAAA
**Lizard**	AAAACC**CG**GGGTTG	GGCGCG**C**AA**A**GAAA
**Turtle**	AAAACC**CG**GGGTTG	GGAGCT**A**AG**A**AAAA
**Chicken**	AAAACC**CG**GGGTTG	GGAGCT**A**AG**A**AAAA
**Duck**	AAAACC**CG**GGGTTG	GGAGCT**A**AG**A**AAAA
**Flycatcher**	AAAACC**CG**GGGTTG	GGAGCT**A**AG**A**AAAA
**Zebra finch**	AAAACC**CG**GGGTTG	GGAGCT**A**AG**A**AAAA
**Frog**	AAAACG**TG**GGGTTG	GGAGCT**A**AG**A**AAAA
**Coelocanth**	AAAACG**CG**GGGTTG	GGAGCT**A**AG**A**AAAA
**Spotted gar**	AAAACG**T-**GGGCTG	GGAGCT**C**AG**C**AAAA
**Zebrafish**	AAAACA**T-**GAGTTG	GGAGCT**C**AG**C**AAAA
**Fugu**	AAAACG**T-**GGGTTG	GGAGCT**C**AG**C**AAAA
**Stickleback**	AAAACG**T-**GGGTTG	GGAGCT**C**AG**C**AAAA
**Cod**	AAAACC**C-**GGGTTG	GGAGCT**C**TG**C**AAAA
**Platyfish**	AAAACA**T-**GGGTTG	GGAGCT**C**AG**C**AAAA
**Tilapia**	AAAACG**T-**GGGTTG	GGAGCT**C**AG**C**AAAA
**Elephant shark**	AAAACC**CC**GGGTTG	GGAGA-**A**AG**A**AAAA
